# Prognostic significance of neutrophil-to-lymphocyte ratio in cervical cancer: A systematic review and meta-analysis of observational studies

**DOI:** 10.18632/oncotarget.15157

**Published:** 2017-02-06

**Authors:** Qi-Tao Huang, Qian-Qian Man, Jia Hu, Yi-Lin Yang, Yue-Mei Zhang, Wei Wang, Mei Zhong, Yan-Hong Yu

**Affiliations:** ^1^ Division of Obstetrics and Gynecology, Nanfang Hospital, Southern Medical University, Guangzhou, 510515, China; ^2^ Southern Medical University, Guangzhou, 510515, China

**Keywords:** cervical cancer, inflammatory, neutrophil, lymphocyte, prognosis

## Abstract

**Background and aims:**

The prognostic role of neutrophil-to-lymphocyte ratio (NLR) in cervical cancer are controversial. We conducted this meta-analysis to obtain a more accurate assessment of prognostic significance of NLR in cervical cancer.

**Results:**

A total of 9 studies, consisting of 2,804 patients, were selected in this meta-analysis. Our pooled results showed that high pre-treatment NLR level was significantly associated with poorer overall survival (HR: 1.88, 95% CI 1.30–2.73) and shorter progression free survival (HR 1.65, 95% CI 1.18–2.29). Additionally, increased NLR was also significantly correlated with tumor size (OR 2.05, 95% CI 1.14–3.65), advanced FIGO stage (OR 2.12, 95% CI1.28–3.49) and lymph node involvement (OR 2.24, 95% CI 1.65–3.04).

**Materials and Methods:**

We conducted a systematic literature search using the electronic databases PubMed, Web of Science, and Embase up to May 2016.Statistical analysis was performed using Stata 10.0.

**Conclusions:**

Elevated pretreatment NLR could serve as a predicative factor of poor prognosis for cervical cancer patients.

## INTRODUCTION

Cervical cancer is the second most common female malignancy, accounting for 500,000 new cases and over 200,000 deaths worldwide annually [1, 2]. Although tumor size, lymph node status, International Federation of Gynecology and Obstetrics (FIGO) staging criteria as well as pretreatment hemoglobin level were reported to be independent prognostic factors for cervical cancer [3–6], these factors are not routinely used in clinical settings due to insufficient specificity and sensitivity among patients. To further increase predictive accuracy, more prognostic parameters are still warranted.

Neutrophil to lymphocyte ratio (NLR), is a marker for evaluating the systemic potential balance between neutrophil-dependent pro-tumor inflammation and lymphocyte-associated anti-tumor immune response [7–8]. A higher level of NLR could represent a trend towards increased pro-tumor inflammation and decreased anti-tumor immune capacity.

Accumulating evidence demonstrates that NLR has prognostic significance in patients with various types of cancers [9–16]. Recently, several studies assessed the prognostic significance of NLR in patients with cervical cancer [17–25]. However, the prognostic significance of NLR in cervical cancer remained controversial. To clarify this issue, we performed this systematic review and meta-analysis to obtain a more reliable assessment of prognostic significance of NLR in patients with cervical cancer.

## RESULTS

### Selection and characteristics of included eligible studies

**Figure 1:**
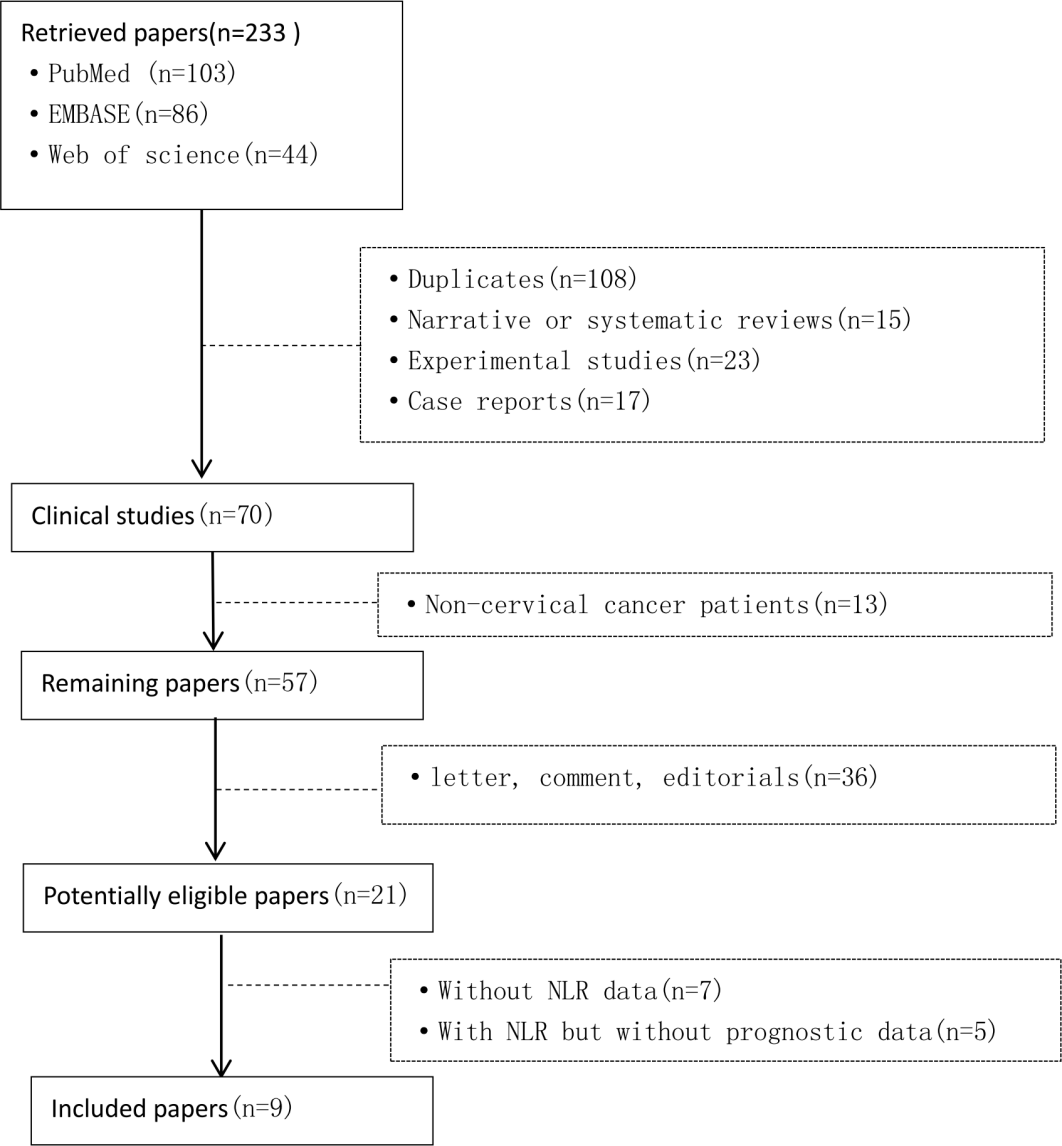
Flow diagram of the studies selection process

A flowchart for the selection of eligible studies is demonstrated in Figure 1. A total of 233 studies was retrieved and screened by title and abstract. 176 studies were excluded after the initial assessment of title and abstract. Among the remaining 57 articles, 36 were further excluded because they were letters, comments, editorials, or reviews. The full texts of the remaining 21 articles were evaluated. A total of 12 full-text articles were excluded, including 7 without available data and 5 without NLR category.

The basic information of the selected studies is summarized in Table 1.

**Table 1:** Characteristics of all identified studies

Study		Year	Country		Treatment		Age (y)		Univariate analysis HR (95%CI)			Multivariate analysis HR (95%CI)		Survival analysis	
Tavares-Murta BM [17]		2010	Brazil		surgery, nonsurgery		48.5 (15–93)		—			—		—	
Onal C [18]		2016	Turkey		nonsurgery		57 (21–86)		—			OS:3.322 (1.905–5.790) PFS:3.579 (2.106–6.082)		OS, PFS	
Nakamura K [19]		2015	Japan		nonsurgery		52.6 (25–78)		OS:2.564 (0.826–7.961)			—		OS	
Mizunuma M [20]		2015	Japan		nonsurgery		65.1 (35–89)		—			OS:2.80 (0.83–9.34) PFS: 1.53 (1.19–1.97)		OS, PFS	
Wang YY [21]		2016	China		nonsurgery		53 (36–80)		—			OS:3.731 (1.082–12.821)		OS, PFS	
Zhang Y [22]		2014	China		surgery		44 (24–78)		—			OS:1.631 (0.968–2.750) PFS:1.799 (1.069–3.028)		OS, PFS	
Lee YY [23]		2012	Korea		surgery, nonsurgery		50 (21–85)		OS:1.19 (1.15–1.24) PFS:1.16 (1.12–1.20)			OS:1.19 (1.13–1.25) PFS:1.13 (1.08–1.18)		OS, PFS	
Zheng RR [24]		2016	China		surgery		49.5 (38.8–60.2)		OS:1.480 (0.995–2.201) DFS:1.481 (0.997–2.201)			—		OS, DFS	
Wang D [25]		2013	China		surgery		42 (21–68)		—			—		OS, PFS	
**Study**	**Sample**			**Duration**		**Follow-up (m) (median/range)**		**NLR cut-off**		**Stage**	**Sampling time**		**Study design**		**NOS**
Tavares-Murta BM [17]	315			1990–2002		> 60		5		CIN, I–IV	pretreatment		retrospective		8
Onal C [18]	235			2006–2014		31.7 (3.7–114.2)		3.03		IB2–IVA	pretreatment		retrospective		9
Nakamura K [19]	32			2005–2014		6.6 (1.4–34.1)		3.95		—	pretreatment		retrospective		7
Mizunuma M [20]	56			2005–2013		14.2 (2–41)		2.5		IB1–IV	pretreatment		retrospective		9
Wang YY [21]	60			2009–2010		58 (7–70)		2		II–III	pretreatment		retrospective		7
Zhang Y [22]	460			2005–2008		69 (6–100)		2.213		I–II	pretreatment		retrospective		8
Lee YY [23]	1061			1996–2007		52.9 (1–181)		1.9		IB1–IVA	pretreatment		retrospective		8
Zheng RR [24]	795			—		62.3 (35.6–89)		2.77		IA–IIB	pretreatment		retrospective		8
Wang D [25]	111			1999–2010		20.6 (0.6–40.6)		2.5		IB2–IIB	pretreatment		retrospective		8

OS: overall survival; PFS: progression-free survival; DFS: disease-free survival;”—”: not reported; Nonsurgery was defined as chemotherapy, radiotherapy or chemoradiotherapy.

### Association of pre-treatment NLR with overall survival

**Figure 2:**
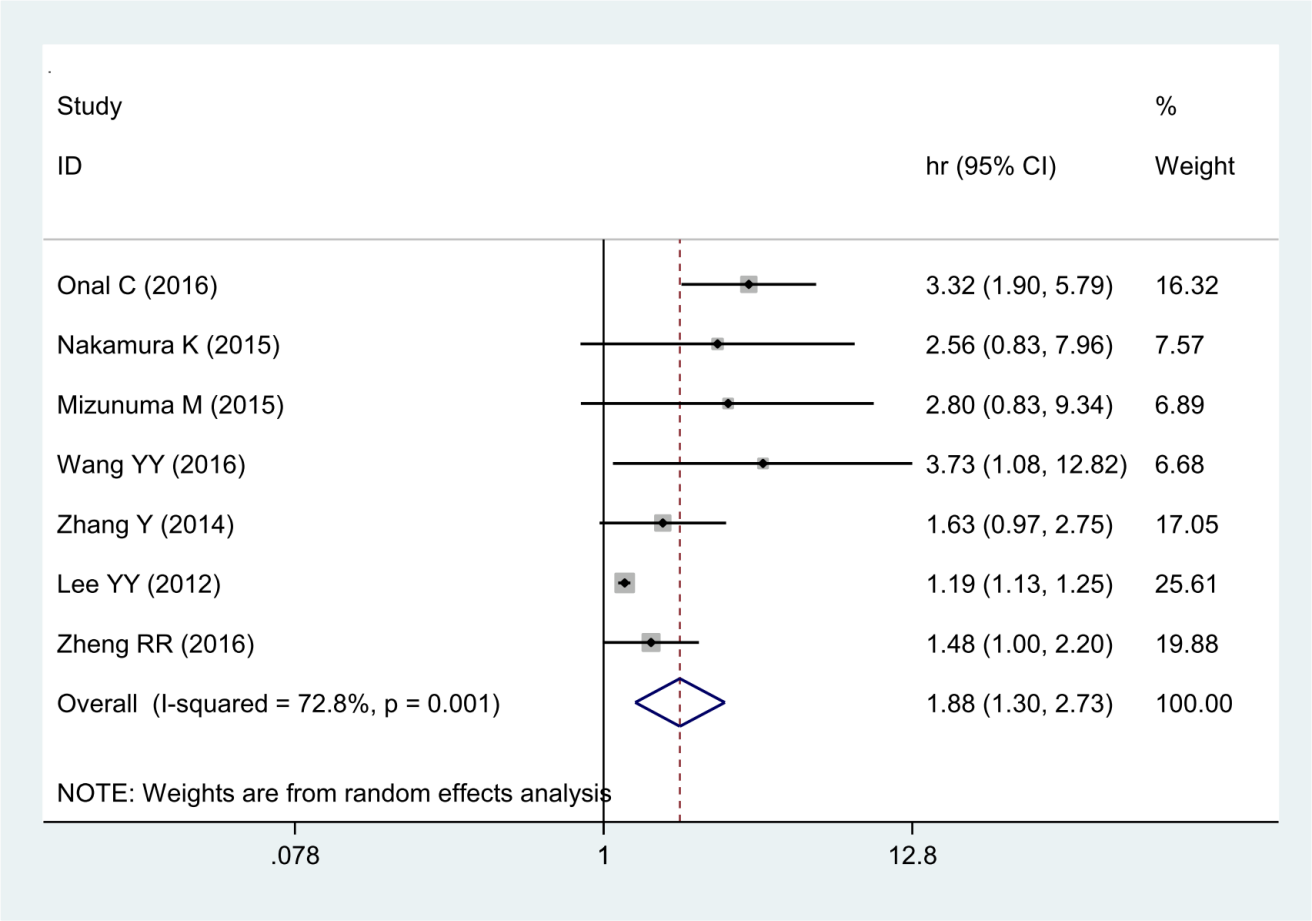
Forest plot of the correlation between NLR and OS in cervical cancer patients

The association between NLR and OS was assessed in 7 studies consisting of 2,804 patients. The pooled estimate indicated a significantly shorter OS in cervical cancer patients with high NLR compared to those with low NLR (HR: 1.88, 95% CI 1.30–2.73) (Figure 2). Significant heterogeneity was observed among these studies (I^2^ = 72.8%, *P <* 0.001).

### Association of pre-treatment NLR with progression free survival

**Figure 3:**
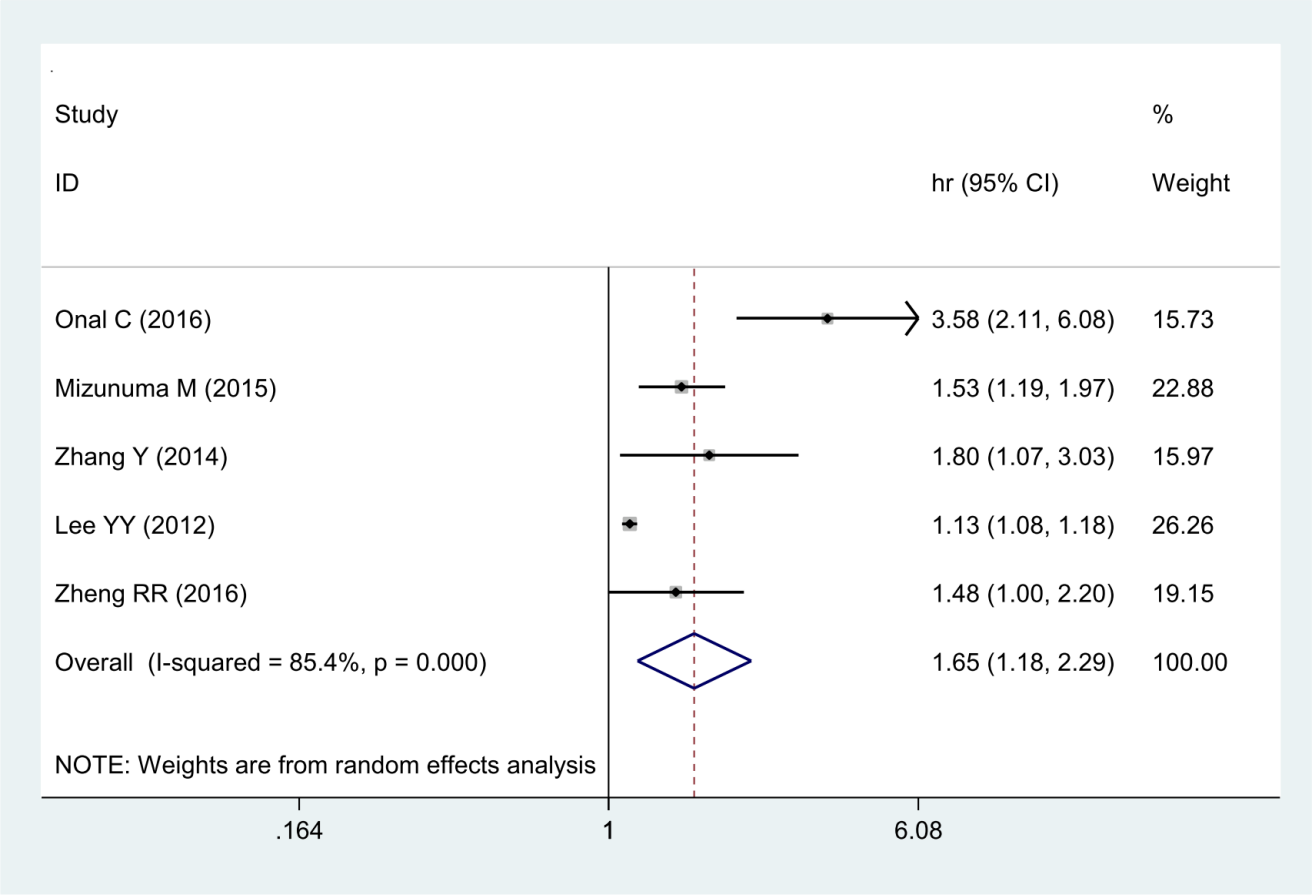
Forest plot of the correlation between NLR and PFS in cervical cancer patients

The association between NLR and PFS was evaluated in 5 studies including 2,607 patients. Those with high pre-treatment NLR had a significantly poorer PFS than those with low NLR (HR 1.65, 95% CI 1.18–2.29) (Figure 3). Significant heterogeneity was observed among these studies (I^2^ = 85.4%, *P <* 0.001)

### Correlations between NLR and clinical-pathological features

**Figure 4:**
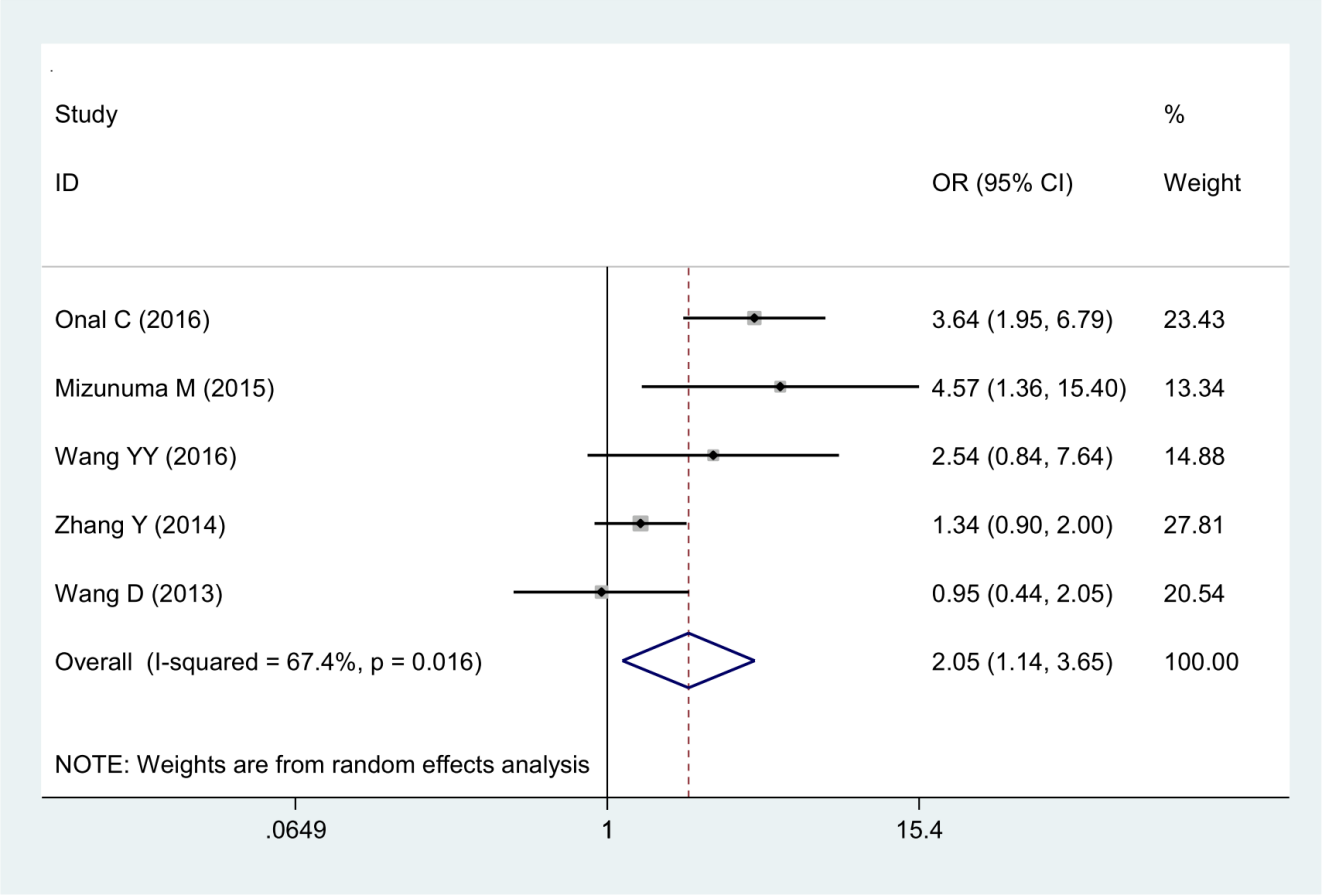
Forest plot of the correlation between NLR and tumor size in cervical cancer patients

**Figure 5:**
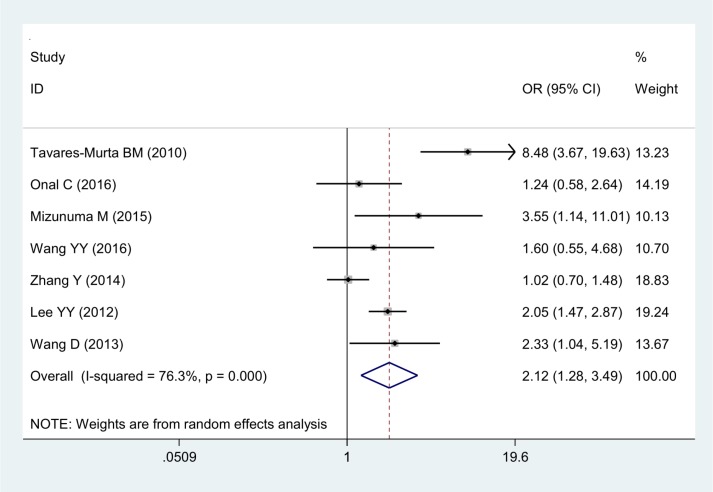
Forest plot of the correlation between NLR and FIGO staging in cervical cancer patients

**Figure 6:**
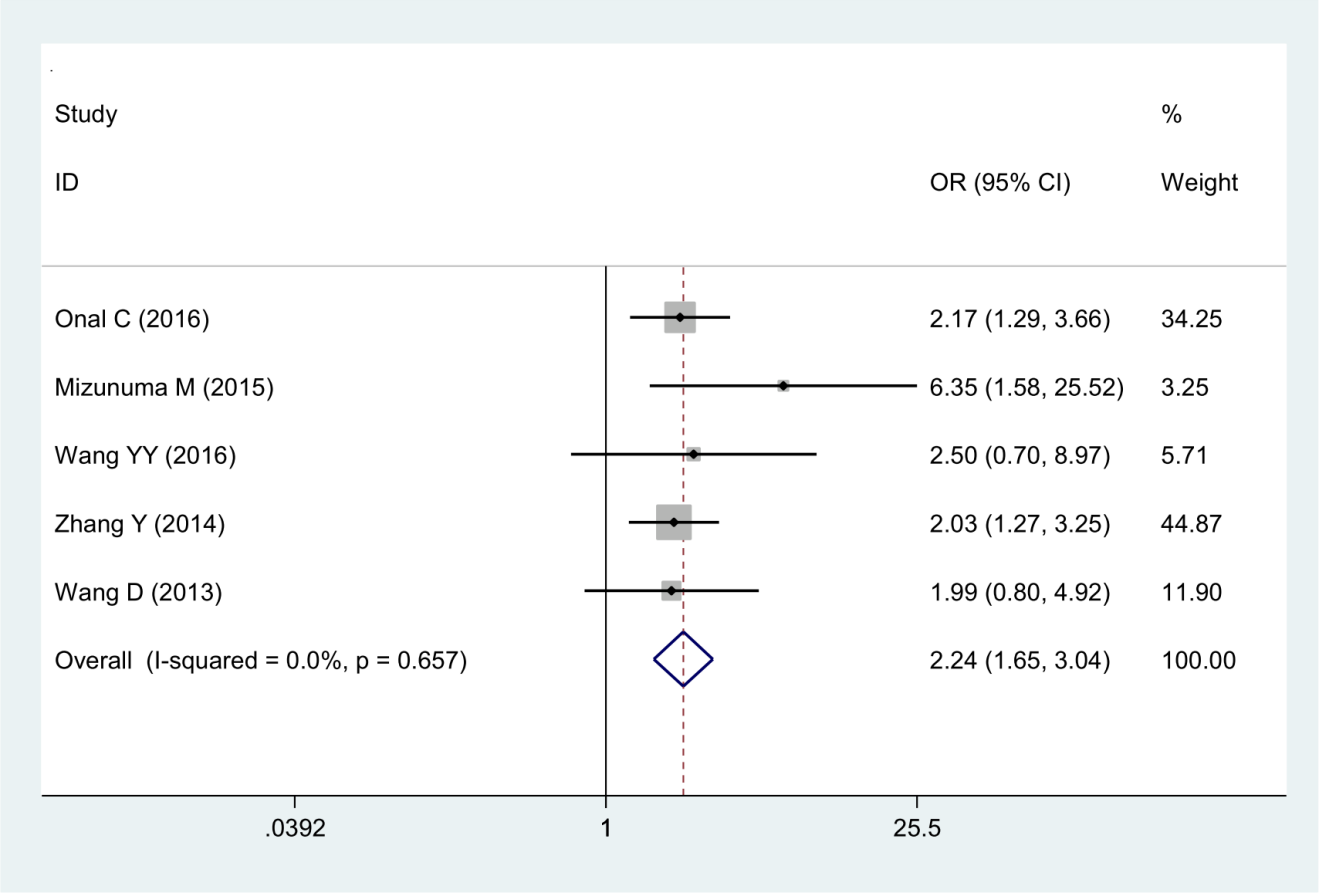
Forest plot of the correlation between NLR and lymph node metastasis in cervical cancer patients

The correlations between NLR and clinical features of cervical cancer are described below. NLR was positively related to tumor size (OR 2.05, 95% CI 1.14–3.65) (Figure 4) and also demonstrated significant correlation with advanced FIGO stage (OR 2.12, 95% CI 1.28–3.49) (Figure 5) with significant heterogeneity. Additionally, NLR was positively associated with lymph node involvement (OR 2.24, 95% CI 1.65–3.04) (Figure 6) without significant heterogeneity.

### Subgroup analysis

Results of subgroup meta-analyses are summarized in Table 2.

**Table 2:** Results of the meta-analysis on predictive value of NLR in cervical cancer

	Overall survival				Progression free survival			
	**N**	**HR**	**LCI**	**HCI**	**N**	**HR**	**LCI**	**HCI**
**Overall**	7	1.88	1.30	2.73	5	1.65	1.18	2.29
**Geographic area**								
1. Asian	6	1.20	1.14	1.26	4	1.38	1.09	1.75
2. non-Asian	1	3.32	1.91	5.79	1	3.58	2.11	6.08
Statistical methods*								
1. Univariate	3	1.19	1.15	1.24	2	1.16	1.12	1.20
2. Multivariate	5	2.05	1.20	3.50	4	1.71	1.14	2.56
**Sample size**								
1. < 300	4	3.17	2.06	4.89	2	2.26	0.99	5.19
2. ≥ 300	3	1.20	1.14	1.26	3	1.32	1.00	1.75
**Treatment**								
1. Surgery	2	1.53	1.12	2.10	2	1.59	1.16	2.18
2. Chemoradiotherapy	4	3.17	2.06	4.89	2	2.26	1.09	5.19
3. Surgery plus chemoradiotherapy	1	1.19	1.13	1.25	1	1.13	1.08	1.18
**FIGO staging**								
1. I–II	2	1.53	1.12	2.10	2	1.59	1.16	2.18
2. III–IV	4	2.31	1.08	4.93	3	1.70	1.06	2.71
3. I–IV	1	2.56	0.83	7.96	0	-	-	-
**NLR standard**								
1. < 3	5	1.20	1.14	1.26	4	1.38	1.09	1.75
2. ≥ 3	2	3.16	1.92	5.20	1	3.58	2.11	6.08
**Follow-up**								
1. < 18	2	2.67	1.17	6.11	1	1.53	1.19	1.97
2. ≥ 18	5	1.77	1.19	2.65	4	1.73	1.08	2.78

* The study (Lee YY 2012) reported HR (95%CI) of both univariate and multivariate analysis results.

### Publication bias analysis

The funnel plots showed a low probability of publication bias (Supplementary Figure 1 and 2). Consistently, the Egger’s and Begger’s regression tests demonstrated little evidence of publication bias for OS (*P* = 0.035; *P* = 0.028) and for PFS (*P* = 0.221; *P* = 0.033), respectively.

### Sensitivity analysis

We performed a sensitivity analysis for every analysis by sequential omission of the individual study. The pooled HRs for OS and PFS were not significantly changed, which suggested the robustness of the results (Supplementary Figures 3 and 4).

## DISCUSSION

Accumulating evidence demonstrated that inflammation exerted an essential role in cancer formation, development, and progression [26–30]. Neutrophils have been considered to be the primary source of circulating VEGF, which play a critical role in tumor-associated angiogenesis through producing many inflammatory cytokines such as tumor necrosis factor, interleukin 1, and providing a favorable micro-environment for tumor [28]. Conversely, lymphocytes exert a critical role in cancer-specific immune response [29]. It has been shown that an increased infiltration of lymphocytes into tumor tissue is associated with good prognosis [30]. In the present study, we found that an elevated NLR was associated with poorer OS and shorter PFS in cervical cancer patients, which was in accordance with the results from studies with several other cancer types.

Previous studies reported that higher pretreatment NLR had a stronger predictive effect in cancer at a more advanced stage [31, 32]. Yodying H et al. [31] evaluated the prognostic role of NLR in esophageal cancer and indicated that NLR was associated with tumor invasion and lymph node metastasis. Meanwhile, Xue TC et al. [32] found that NLR was associated with vascular invasion in hepatic carcinoma. In the present study, our results showed that NLR was positively related to tumor size and significantly correlated with lymph node involvement as well as advanced tumor stage (FIGO). These observations indicated that inflammation severity might significantly affect intrinsic tumor characteristics in patients with cervical cancer.

Heterogeneity was observed in this meta-analysis. This heterogeneity may be partially caused by geographic area, statistical methods, sample size, NLR cut-off value, and the follow-up duration. In order to explore the source of heterogeneity in this meta-analysis, we performed a subgroup analysis. The subgroup analysis results demonstrated that the prognostic value of NLR was unaffected by the confounders mentioned above in the analysis. Moreover, the sensitivity analysis indicated that our results were relatively conclusive.

There are several limitations that need to be addressed in this study. First, most of the studies selected in this meta-analysis were retrospective, observational studies, and no prospective cohort study was identified. Moreover, most studies only contained information of NLR predictive value in the same stages or in the same therapy. Therefore, they may be more susceptible to bias in data analysis and the predictive value of NLR in the same stages and the same therapy need to be explored in the future. Second, a previous systemic review demonstrated that increased NLR predicted poor PFS with prostate cancer only in Asians, but not in Caucasians, which could be attributed to the ethnicity heterogeneity [33]. In this meta-analysis, majority of included articles came from Asian countries. Therefore, our current conclusions may not be suitably applied to other populations. Third, only 9 studies were included and the cut-off value for defining high NLR in each individual study was different, ranging from 1.9 to 5, which could hinder the application of this ratio in the clinical setting. In the future, original research is required to determine the most accurate cut-off value of NLR in cervical cancer patients.

Despite these limitations, our meta-analysis also has some strengths. To our knowledge, this meta-analysis is the first to evaluate the prognostic role of a pretreatment peripheral blood NLR in cervical cancer. Moreover, our results showed a significantly positive correlation between NLR and the clinical features of cervical cancer, such as tumor size, FIGO stage, and lymph node involvement. Thus, NLR could have a wider clinical application regarding the prognostic assessment of cervical cancer, may be useful in stratifying patients, and in determining individual treatment plans in the future.

In conclusion, our meta-analysis of currently available clinical evidence demonstrated that NLR could serve as a promising prognostic marker of cervical cancer, because it is available from routine blood tests in daily clinical practice, which are convenient, low cost, and reproducible. In addition, considering that NLR level was associated with prognosis of cervical cancer, it would be interesting to explore whether decreasing the inflammatory conditions, such as lowing NLR level could serve as an adjuvant therapy and therefore prolong the survival of cervical cancer patients in the near future.

## MATERIALS AND METHODS

This systematic review and meta-analysis was performed following the guidance provided in the Cochrane Handbook and was reported according to the Meta-analysis of Observational Studies in Epidemiology (MOOSE) guidelines [34].

### Search strategy

We conducted a systematic literature search using the electronic databases PubMed, Web of Science, and Embase up to May 2016. Search terms included “neutrophil to lymphocyte ratio”, “NLR”, “cervix”, “cervical” and “tumor, cancer, neoplasm, carcinoma or malignancy”. The titles and abstracts of potential references were scanned carefully to exclude irrelevant articles. The remaining studies were assessed to identify the topic of interest, and full texts were then reviewed comprehensively.

### Selection criteria

A study was included if it met the following criteria: (1) included patients with cervical cancer diagnosed histopathologically; (2) provided pre-treatment and/or post-treatment NLR and cut-off values, (3) evaluated the associations between pre-treatment and/or post-treatment NLR and survival outcomes. Exclusion criteria were (1) review articles, editorial comments, letters, expert opinion, conference abstracts, or case reports; (2) insufficient data for estimating hazard ratios (HRs) and 95% confidence intervals (CIs); or (3) full text unavailable and non-English article.

All assessments were conducted independently by two reviewers to assure accuracy of inclusive studies. Multivariate data were preferred to univariate data if both were provided. However, univariate data were acceptable if no multivariate results were presented.

### Data extraction

Two investigators independently gathered information from each eligible study. Data was extracted as follows: surname of first author, study country, year of publication, sample size, cancer stage, treatment method, cut-off value defining elevated NLR and HRs with 95% CI for overall survival (OS) and progress-free survival (PFS)/recurrence-free survival (RFS). Disagreements in data extraction were resolved through discussion.

### Assessment of methodological quality

Two independent investigators assessed the quality of each included study using the Newcastle-Ottawa Quality Assessment Scale (NOS) [35]. On a score scale from 0 to 9, a study with 7 or more stars was considered as high-quality.

### Statistical analysis

The HR and corresponding 95% CI were used to evaluate the prognostic efficiency of NLR on cervical cancer. In addition, the relationship between NLR and clinical-pathological features were reported as ORs and 95% CIs. Cochran’s *Q* test and Higgins I-squared statistic were adopted to test the heterogeneity of pooled data. *I^2^ < 50%* and *p > 0.1* indicated no significant heterogeneity and fixed-effects model was applied to combine the effective value [36]. Otherwise, a random-effects model was adopted. All statistical analyses were performed using STATA 12.0 software (StataCorp LP, TX, USA).

Subgroup analyses were performed to investigate the associations of NLR with clinical features in relation to geographic area, statistical methods, sample size, cancer stage, lymph node involvement, NLR cut-off value, and follow-up duration. Moreover, a sensitivity analysis was performed to examine the robustness of the pooled results.

## SUPPLEMENTARY MATERIALS FIGURES AND TABLES


