# Supplementary material for: Prognostic significance of neutrophil-to-lymphocyte ratio in cervical cancer: A systematic review and meta-analysis of observational studies
Source: Oncotarget. 2017 Feb 6;8(10):16755–64. doi: 10.18632/oncotarget.15157 (PMC5369999; doi:10.18632/oncotarget.15157)
Supplement: Supplementary file 1 [file oncotarget-08-16755-s001.pdf]

Prognostic significance of neutrophil-to-lymphocyte ratio in cervical cancer: A systematic review and meta-analysis of observational studies

Supplementary Materials

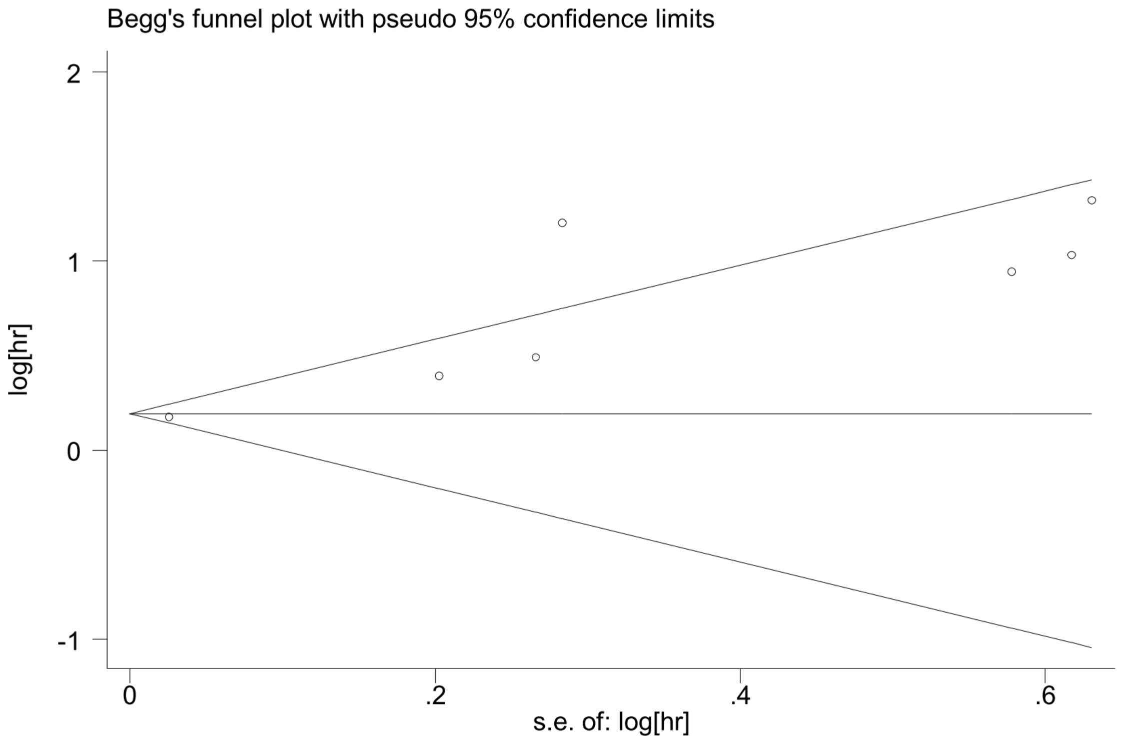

Supplementary Figure 1: Funnel plot of seven studies included in this meta-analysis for OS.

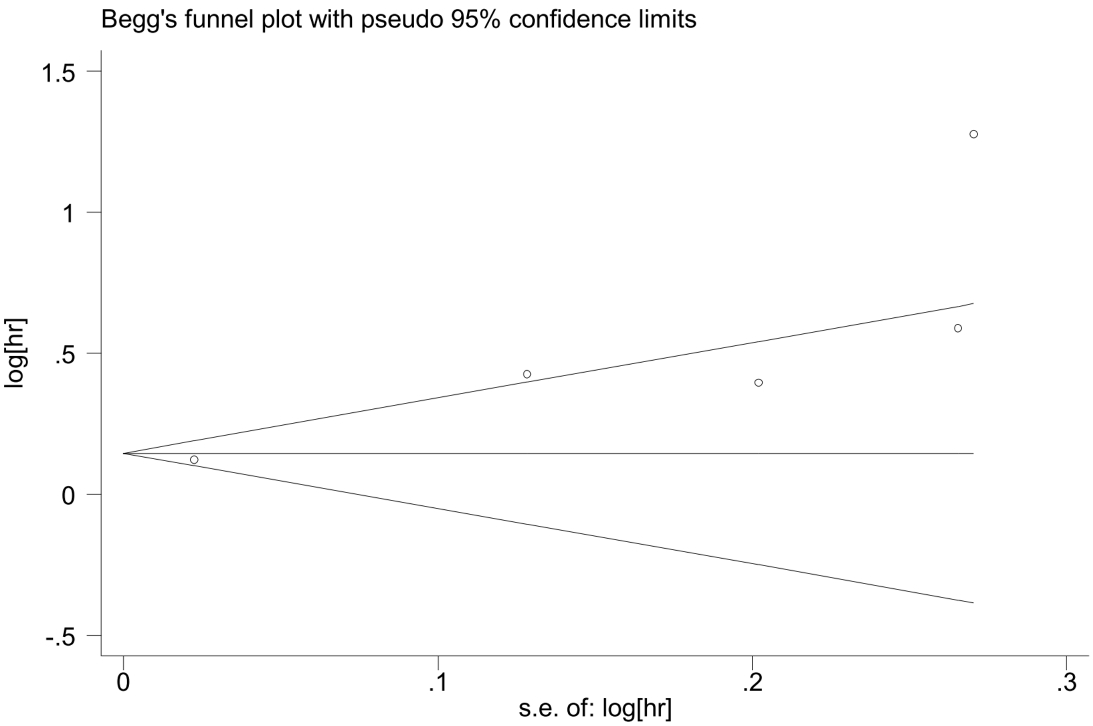

Supplementary Figure 2: Funnel plot of five studies included in this meta-analysis for PFS.

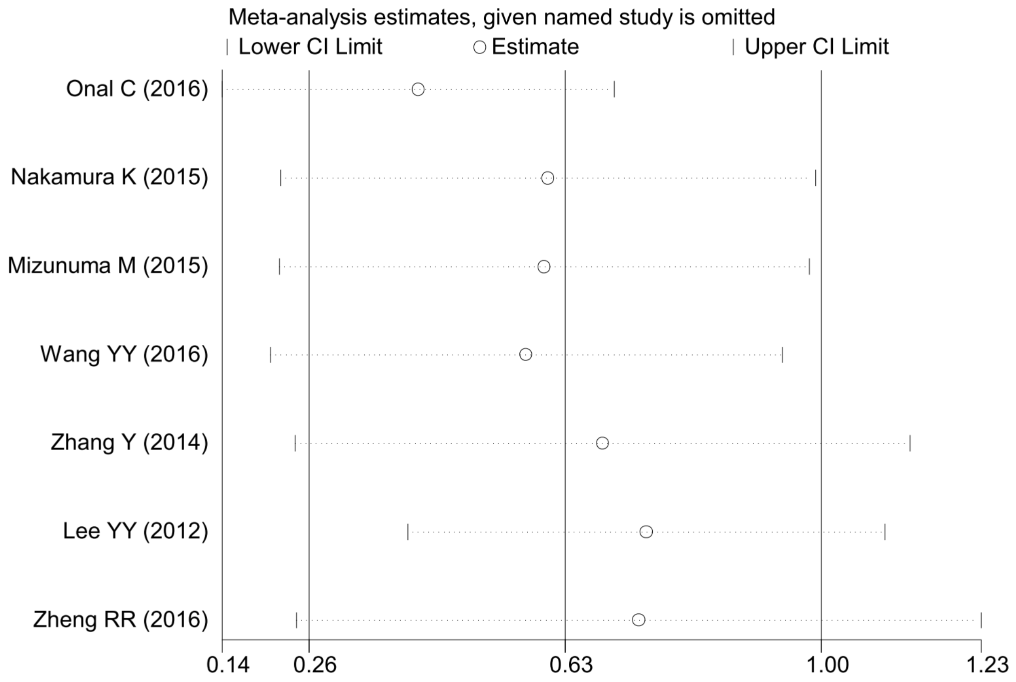

Supplementary Figure 3: Sensitivity analysis of seven studies included in this meta-analysis for OS.

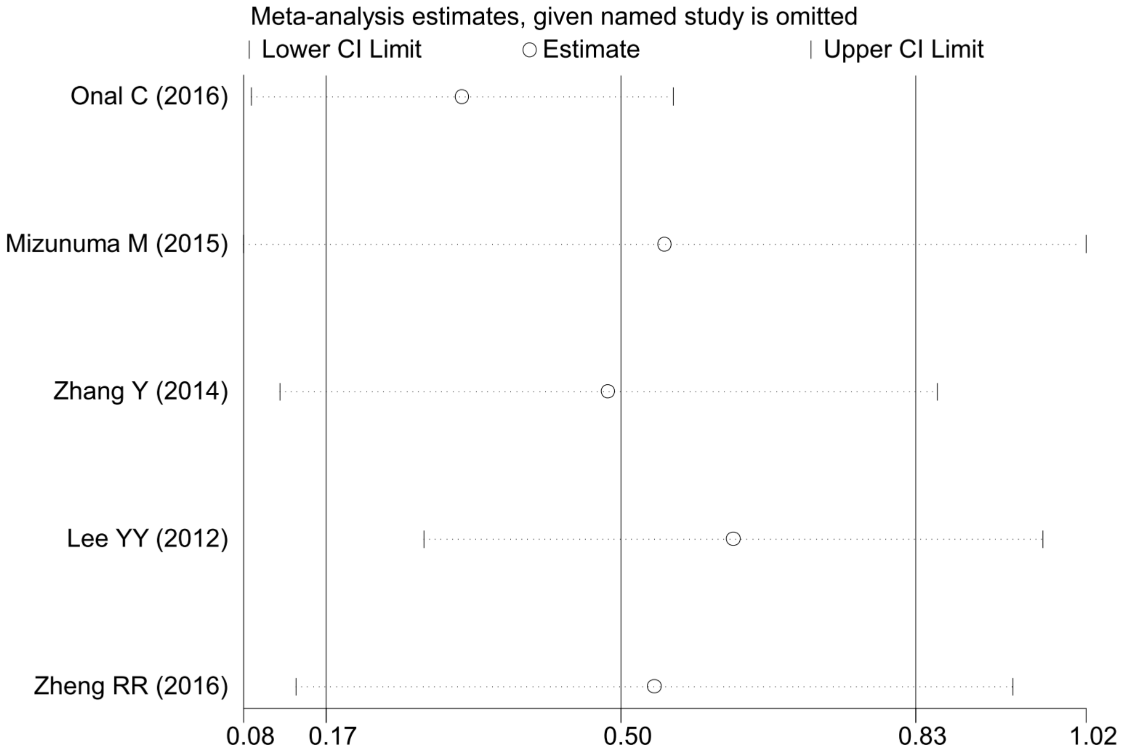

Supplementary Figure 4: Sensitivity analysis of five studies included in meta-analysis for PFS.
